# Global landscape of locally produced alcohol-based handrub in health care settings: a scoping review

**DOI:** 10.1186/s13756-026-01757-0

**Published:** 2026-05-09

**Authors:** Hiroki Saito, Ermira Tartari, Jacopo Garlasco, Didier Pittet, Benedetta Allegranzi

**Affiliations:** 1 Department of Infectious Diseases, Keio University School of Medicine, Shinanomachi, Shinjyuku-Ku, Tokyo Japan; 2 Department of Emergency and Critical Care Medicine, St. Marianna University School of Medicine, Kawasaki, Kanagawa Japan; 3 Faculty of Health Sciences, University of Malta, Msida, Malta; 4 Infection Prevention and Control Technical and Clinical Hub, Department of Integrated Health Services, World Health Organization (WHO), Geneva, Switzerland; 5 Infectious Disease Unit, Department of Diagnostics and Public Health, University of Verona, Verona, Italy; 6 Hospitals and Faculty of Medicine, University of Geneva, Geneva, Switzerland; 7 Communicable Diseases Department, World Health Organization Regional Office for the Eastern Mediterranean, Cairo, Egypt

**Keywords:** Local production, Alcohol-based handrub, Healthcare associated infections, Hand hygiene, Infection prevention and control

## Abstract

**Background:**

Reliable access to alcohol-based handrub (ABHR) is essential for hand hygiene and infection prevention, yet many low- and middle-income countries (LMICs) continue to face supply constraints. A 2011 WHO global assessment demonstrated that WHO-recommended ABHR formulations produced locally at low cost, were well accepted by healthcare workers, but also highlighted persistent barriers, including challenges in procuring ingredients and dispensers and in ensuring adequate quality control. This review aimed to provide an updated global synthesis of evidence on local ABHR production in healthcare settings.

**Methods:**

Following the Joanna Briggs Institute framework and the Preferred Reporting Items for Systematic reviews and Meta-Analyses extension for Scoping Reviews (PRISMA-ScR) guidelines, we systematically searched Embase, Medline, and CINAHL from inception to 19 March 2025. Two reviewers independently conducted title-abstract screening, full-text screening and data extraction. Primary research articles reporting local ABHR production in healthcare settings in LMICs were eligible for data extraction and descriptive synthesis.

**Results:**

Of 2343 articles screened, 31 studies from 19 countries were included (2006–2023). Over half (n = 18, 58%) were conducted during the COVID-19 pandemic; 22 described health-facility production and 9 described factory-level manufacturing. Of the 22 health-facility production studies, 12 (55%) used WHO Formulation 1 (ethanol-based) or modifications thereof. Pharmacists most commonly led production at the health-facility level. Only 6% (two articles) reported the source of alcohol, and less than half evaluated efficacy or organoleptic properties (13 and 12, respectively). Most funded studies relied on high-income-country (HIC) grants (17 of 24, 71%).

**Conclusions:**

Local ABHR production remains infrequently reported in the literature, although publications increased during the COVID-19 pandemic. Heavy reliance on short-term, HIC-funded initiatives raises concerns about the long-term scalability and sustainability of local ABHR production in LMICs. Strengthening national regulatory capacity, quality-control laboratories, and sustainable financing is critical to maintain safe ABHR access beyond pandemic contexts, for resilient national supply chains and in-country quality control capacity.

**Supplementary Information:**

The online version contains supplementary material available at 10.1186/s13756-026-01757-0.

## Background

Alcohol-based handrub (ABHR) is a cornerstone of hand hygiene in health care and is included in the World Health Organization (WHO) Essential Medicines List [1]. In its 2009 Guidelines on Hand Hygiene in Health Care, WHO identified “system change”, ensuring reliable access to essential hand hygiene products and infrastructure, as a key component of the Multimodal Hand Hygiene Improvement strategy [2]. To support this, WHO developed guidance for local ABHR production, recommending two formulations (ethanol-based Formulation 1 and isopropyl alcohol-based Formulation 2) to facilitate affordable, safe, and context-appropriate supply [3].

Despite these efforts, access to safe and reliable hygiene products remains a major barrier in many settings. In 2019, WHO and United Nations Children’s Fund (UNICEF) reported that one in four healthcare facilities globally, and nearly half in sub-Saharan Africa, lacked basic water services [4], jeopardizing progress toward the United Nation’s Sustainable Development Goal (SDG) 6 (Clean Water and Sanitation), and SDG 3 (Good Health and Well-Being) [5]. The COVID-19 pandemic further strained these systems, leading to shortages of safe water and ABHR and the widespread circulation of substandard products [6–8], Water, sanitation and hygiene (WaSH), infection prevention and control (IPC) and universal health coverage (UHC) are closely interdependent, underpinning local resilience, whose priority was highlighted in the post-pandemic context.

In 2011, WHO global assessment of 34 healthcare facilities across 29 countries, including 23 low- and middle-income countries (LMICs) [9], demonstrated that WHO-recommended ABHR formulations could be produced locally at low cost and with good tolerability among healthcare workers, but also identified persistent challenges in procuring ingredients and dispensers and ensuring adequate quality control. More than a decade later, and despite the availability of WHO guidance and early successful pilots, no global synthesis has mapped how and where local ABHR production has been implemented. WHO has recently identified the need to understand approaches that support sustained system change as a high-priority research area for hand hygiene in health care [10, 11].

To address these gaps, we conducted a scoping review to provide an up-to-date global assessment of local ABHR production in healthcare settings, describe geographic and temporal trends, and identify factors influencing sustainability and quality assurance.

## Methods

This scoping review followed the Joanna Briggs Institute (JBI) methodology for scoping reviews and is reported in accordance with the Preferred Reporting Items for Systematic Reviews and Meta-Analyses extension for Scoping Reviews (PRISMA-ScR) [12, 13]. A scoping review design was selected to map the extent, range, and nature of the evidence on local ABHR production, recognizing the heterogeneity of study designs and implementation contexts.

### Eligibility criteria

We included primary research articles reporting the local production of ABHR within healthcare settings in LMICs. Country income levels were determined according to the World Bank classification system applicable at the time of each study’s publication [14]. Local production was defined as ABHR manufactured within the country where it was used, including production at facility, district, or national factory level.

We excluded studies that described ABHR production outside healthcare settings (e.g., community settings), studies conducted exclusively in high-income countries (HICs), and non-original publications (reviews, commentaries, letters), as well as animal studies, and non-English language publications. The review protocol was pre-registered and published in an open-source platform previously (Open Science Registries (OSF), 10.17605/OSF.IO/3KA54) [15].

### Information sources

A literature search was conducted in Embase (Elsevier), Medline (Ovid), and CINAHL (EBSCOhost) from database inception to 19 March 2025. Reference lists of included studies and relevant WHO or institutional reports were reviewed to identify additional eligible publications. Grey literature was not systematically searched due to resource constraints and the focus on peer-reviewed evidence.

### Search strategy

Search strategies were developed in collaboration with an experienced medical librarian and combined controlled vocabulary (e.g., MeSH, Emtree) with free-text terms related to hand sanitizer, alcohol-based handrub, local production, and manufacturing. No date or study-design limitations were applied. Full search strategies for each database, including record counts, are provided in Appendix 1.

### Screening and selection

**Fig. 1:**
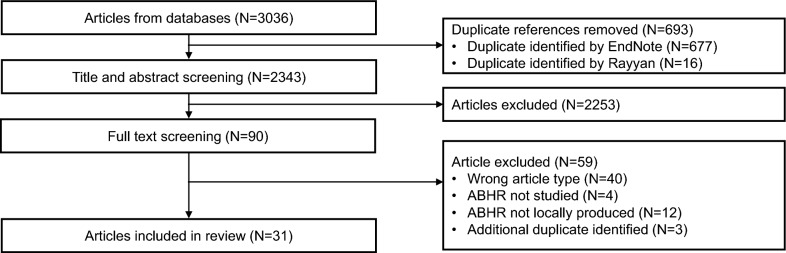
PRISMA flow diagram of a scoping review on local alcohol-based hundrub production

All records were imported into EndNote 21 for deduplication before being uploaded to Rayyan (https://www.rayyan.ai/) for screening. Two reviewers independently screened titles and abstracts, followed by full-text assessment against the inclusion criteria (HS, ET and JG). Disagreements were resolved through discussion or adjudication by a third reviewer. Full-text articles meeting eligibility criteria were included for data extraction. Reasons for exclusion at the full-text stage were documented. The overall selection process is presented in a PRISMA-ScR flow diagram (Fig. 1).

### Data charting

Data were extracted independently by two reviewers using a pre-defined and pilot-tested charting form. Extracted variables included: study identifiers (country, year of publication, study period, study design), linkage to the COVID-19 pandemic, ABHR formulation type, production setting (facility-level or factory-level), professional cadres involved in production, evaluation of ABHR characteristics (e.g., efficacy, acceptability/tolerability, organoleptic properties), and funding sources. Any discrepancies in extracted data were resolved through consensus or third-reviewer arbitration. Consistent with JBI guidance, no critical appraisal of study quality was undertaken.

### Synthesis of results

Extracted data were synthesized descriptively to map geographical distribution, temporal trends, production modalities, and evaluation practices related to local ABHR production. Frequencies and proportions were calculated using Microsoft Excel, and findings were summarized narratively and in tabular form.

## Results

The literature search was conducted on March 19, 2025, yielding 3036 articles identified from the databases. After removing duplicates of 693 articles, a total of 2343 articles were reviewed for title-abstract screening, followed by 90 articles for full-text screening. Subsequently, 31 articles were selected for data extraction and synthesis (Fig. 1).

### Characteristics of the eligible articles in the scoping review

**Fig. 2:**
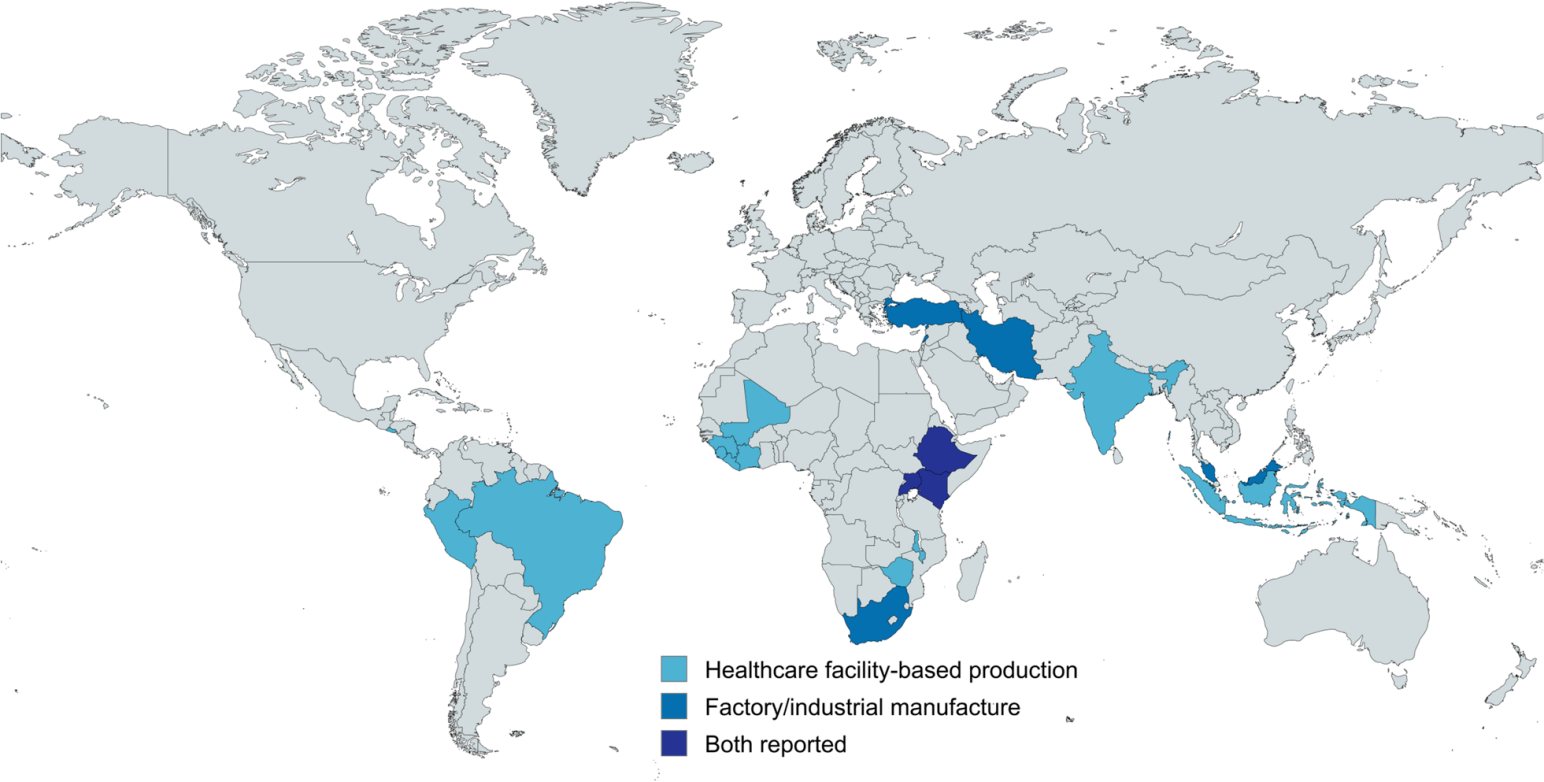
Countries reporting local alcohol-based hundrub production

**Fig. 3:**
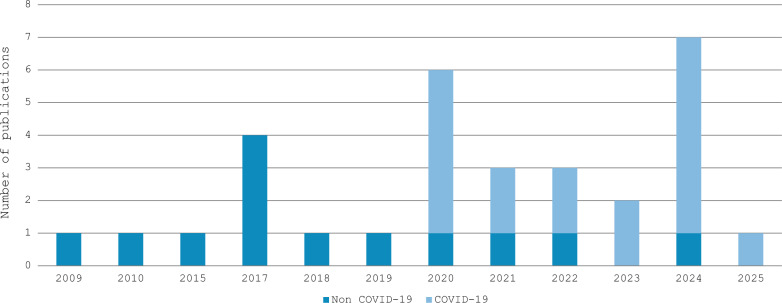
Number of publications by year of the eligible articles, categorized by the COVID-19 pandemic related vs not

The characteristics of the 31 eligible articles included in the scoping review was summarized in Table 1. The included studies spanned 2006–2023 across 19 countries and six WHO regions: Africa accounted for two thirds (n = 21, 67.7%), followed by Asia (n = 6, 19.4%) (Fig. 2). Nine articles were published before 2020 whereas 22 were published in 2020 or later: eighteen articles (n = 18, 58.1%) were relevant to the COVID-19 pandemic (Fig. 3). Seventeen articles (54.8%) were single-year studies. Approximately half of the articles (n = 16, 51.6%) were non-interventional studies, either cross-sectional design (n = 10) or descriptive nature/institutional report (n = 6), and the rest 15 articles were quasi-experimental studies. ABHR was produced at health-facility level in 22 articles (71.0%) and manufactured at factories in nine articles (29.0%), respectively. Among the articles of ABHR production at health-facility level, the WHO Formulation 1 (ethanol-based) including modifications was most commonly produced (12 out of 22, 54.5%), and pharmacists were the main profession in charge of the production (11 out of 11 articles reporting the profession, 100%), followed by laboratory technicians. The source of alcohol was rarely reported (two out of 31, 6.5%). The majority of the funded studies (17 out of 24 articles, 70.8%) were supported by grants from HICs.

**Table 1:** Characteristics of the eligible studies of a scoping review on local alcohol-based handrub production

Author	Country	Study year	Impact of COVID-19	Study design	ABHR formulation	ABHR format	Efficacy evaluation	Organoleptic evaluation	Organoleptic characteristics	Health Facility produced vs Factory manufactured	Professions of the staff producing ABHR	Funding
Nigro et. al. [28]	Brazil	2020	Yes	Descriptive report of institutional experience	ABHR containing 70% ethanol concentration, according to the Brazilian Pharmacopeia National Form	Rinse, Gel	Yes	Yes	Appearance, Texture	Facility	Pharmacists, Pharmacy technicians	no specific funds
Müller et. al. [29]	Côte d'Ivoire	2018–2020	Yes	Quasi-experimental	WHO formulation 1	Rinse	Yes	No	n/a	Facility	Pharmacists, Laboratory technicians, Hygiene experts	Governmental (HIC)
Caniza et. al. [30]	El Salvador	2007	No	Quasi-experimental	ABHR following the local standard procedure (62% ethanol concentration, citrus aroma added)	Gel	No	Yes	Smell, Texture	Facility	Pharmacist	Philanthropic (HIC)
Pfäfflin et. al. [31]	Ethiopia	2016	No	Quasi-experimental	WHO formulations (details unknown)	not specified	No	Yes	Skin tolerability	Facility	not specified	Governmental (HIC)
Gebremicael et. al. [32]	Ethiopia	2018–2019	No	Quasi-experimental	WHO formulation 1	not specified	Yes	No	n/a	Facility	not specified	Governmental (HIC), Philanthropic (HIC)
Manaye et. al. [33]	Ethiopia	2020	Yes	Cross-sectional	Various types of ABHR from local vendors tested for efficacy (mostly ethanol based)	not specified	Yes	No	n/a	Factory	n/a (Commercial entities)	Academia (LIC)
Selam et. al. [34]	Ethiopia	2020	Yes	Cross-sectional	not specified	not specified	No	No	n/a	Facility	not specified	Governmental (LIC)
Selam et. al. [35]	Ethiopia	2020	Yes	Cross-sectional	not specified	not specified	No	No	n/a	Facility	not specified	no specific funds
Selam et. al. [23]	Ethiopia	2022	Yes	Cross-sectional	Various types of ABHR in market tested for content and efficacy	not specified	Yes	Yes	Appearance, Smell	Factory	n/a (Commercial entities)	Academia (LIC)
Müller et. al. [36]	Guinea	2017–2019	No	Quasi-experimental	WHO formulations 1	not specified	Yes	Yes	Appearance, Skin tolerability, Acceptance among HCWs	Facility	Pharmacists	Governmental (HIC)
Müller et. al. [37]	Guinea	2017–2019	No	Quasi-experimental	not specified	not specified	No	No	n/a	Facility	Pharmacists	Governmental (HIC)
Sharma et. al. [38]	India	2009–2011	No	Quasi-experimental	Modified WHO formulation: 95% ethanol (lower by 1% compared to WHO formulation 1), 3% hydrogen peroxide, glycerol, water, and rose extract	not specified	Yes	Yes	Appearance, Skin tolerability	Facility	not specified	Governmental (HIC)
Khurana et. al. [39]	India	2020	Yes	Descriptive report of institutional experience	WHO formulations 1 and 2 (isopropyl alcohol)	not specified	Yes	No	n/a	Facility	not specified	not reported
Tulsawani et. al. [40]	India	not specified	Yes	Descriptive report of institutional experience	73% isopropyl alcohol-based, herbal extracts (Aloe vera, Azadirachta indica, Citrus limon, Zingiber officinale/Ocimum sanctum)	not specified	Yes	No	n/a	Facility	not specified	Governmental (MIC)
Putri et. al. [41]	Indonesia	2014	No	Cross-sectional	WHO formulation 1	not specified	Yes	No	n/a	Facility	not specified	no specific funds
Rafizadeh et. al. [42]	Iran	2020	Yes	Cross-sectional	Various types of ABHR in market tested to meet standards by WHO and Food and Drug Administration guidelines	not specified	No	No	n/a	Factory	n/a (Commercial entities)	Academia (MIC)
Ndegwa et. al. [43]	Kenya	2012–2014	No	Quasi-experimental	ABHR with 75% alcohol concentration, using 99.8% isopropyl alcohol, 6% hydrogen peroxide, and 99% glycerol	not specified	No	Yes	Smell, Skin tolerability, Feeling on skin	Facility	Pharmacists	Governmental (HIC, MIC)
Ochwoto et. al. [44]	Kenya	2015	No	Cross-sectional	Various types of ABHR in market and hospitals tested for efficacy (mostly ethanol or isopropyl alcohol based)	Rinse, Gel	Yes	Yes	Appearance, Feeling on skin, Ease of use	Factory	n/a (Commercial entities)	Governmental (HIC, MIC)
Saab et. al. [24]	Lebanon	2021–2022	Yes	Cross-sectional	Various types of ABHR in market tested for content and quality	Gel, Spray	No	No	n/a	Factory	n/a (Commercial entities)	Academia (MIC)
Bausch et. al. [45]	Liberia, Guinea	2014–2016	No	Descriptive report of institutional experience	WHO formulation 1 (ethanol purchased from a local commercial entity)	not specified	No	No	n/a	Facility	Pharmacists, Laboratory technicians	Academia (HIC), Governmental (HIC)
Kachingwe et. al. [46]	Malawi	2020	Yes	Descriptive report of institutional experience	Modified WHO formulation 1	Gel	Yes	Yes	Texture, Skin tolerability, Feeling on skin	Facility	Pharmacists	not reported
Lee et. al. [47]	Malaysia	2021	Yes	Descriptive report of institutional experience	three ABHRs, ethanol-based (70–83% ethanol)	Gel	No	Yes	Appearance, Smell, Texture, Skin tolerability, Drying effect, Speed of drying	Factory	n/a (Commercial entities)	no specific funds
Allegranzi et. al. [18]	Mali	2006–2008	No	Quasi-experimental	WHO formulation 1 (ethanol produced in Mali from sugar cane)	not specified	No	Yes	Appearance, Smell, Skin tolerability	Facility	Pharmacists	UN agency, Academia (HIC), Governmental (HIC)
Fofanah et. al. [48]	Sierra Leone	2022–2023	Yes	Quasi-experimental	WHO formulation 1	not specified	No	Yes	Appearance, Smell, Texture, Skin tolerability, Feeling on skin, Ease of use, Drying effect, Speed of drying	Facility	Pharmacists, Physicians, Nurses	Governmental (HIC)
de Bruin et. al. [49]	South Africa	2020	Yes	Cross-sectional	Various types of ABHR in market tested for content, quality and safety (ethanol-based, propanol-based, or both)	Rinse, Gel	No	No	n/a	Factory	n/a (Commercial entities)	UN agencies
Berkkan et. al. [25]	Turkey	2020	Yes	Cross-sectional	Various types of ABHR in market tested for alcohol content (colognes containing ethanol 37.9% to 98.9% w/w)	Rinse	No	No	n/a	Factory	n/a (Commercial entities)	not reported
Ishida et. al. [21]	Uganda	2018–2022	Yes	Quasi-experimental	not specified	not specified	No	No	n/a	Facility	not specified	Governmental (HIC)
Saito et. al. [19]	Uganda	2014–2015	No	Quasi-experimental	ABHR locally made by a commercial entity, meeting international standards (Good Manufacturing Practice) (76.9 to 81.4 vol% ethanol, made from sugar care)	Rinse	No	No	n/a	Factory	n/a (Commercial entity)	Commercial
Tusabe et. al. [50]	Uganda	2019–2020	Yes	Quasi-experimental	WHO formulations 1 and 2 (isopropyl alcohol)	Rinse	No	No	n/a	Facility	Pharmacists, Laboratory technicians	Governmental (HIC)
Tusabe et. al. [22]	Uganda	2018–2023	Yes	Quasi-experimental	not specified	not specified	No	No	n/a	Facility	not specified	Governmental (HIC)
Gudza-Mugabe et. al. [51]	Zimbabwe	2015	No	Quasi-experimental	WHO formulation 1	not specified	Yes	No	n/a	Facility	not specified	Governmental (HIC)

ABHR, alcohol-based handrub; HIC, high-income country; LIC, low-income country; MIC, middle-income country; UN, United Nations; WHO, World Health Organization

Efficacy evaluation was reported for 13 articles (41.9%): of these, three (23.1%) explicitly mentioned attaining or overcoming the recommended threshold of a 5-log reduction in bacterial load, for bacterial agents commonly found as human colonizers or pathogens (*Escherichia coli*, *Staphylococcus aureus*, *Pseudomonas aeruginosa*, and/or *Enterococcus hirae*) [16]. Seven (53.8%) reported a significant reduction in bacterial load for pathogens belonging to Enterobacterales*, Staphylococci* and/or *Micrococci*, while the remaining three articles (23.1%) simply declared the efficacy of the locally produced formulation without reporting precise figures.

Organoleptic evaluation was conducted in 12/31 articles (38.7%): among these, the vast majority assessed skin tolerability/feeling on skin (9/12, 75.0%) and appearance (8/12, 66.7%) of the ABHR produced, around half of the studies evaluated smell (6/12, 50.0%) and texture (5/12, 41.7%), while only 2 studies (16.7%) assessed additional parameters such as ease of use and drying speed.

## Discussions

The scoping review provides a global synthesis in more than a decade of local ABHR production within healthcare settings in LMICs. Thirty-one studies from 19 countries were identified, with more than half published during the COVID-19 pandemic. Although both facility-level production and factory-level manufacturing were reported, the overall volume of evidence remains limited relative to global need. This suggests that sustained access to ABHR, an essential IPC imperative, likely continues to pose challenges in many LMICs. The marked increase in production during the COVID-19 pandemic, reflects both the adaptive capacity of local systems under crisis conditions and the vulnerability of supply chains dependent on emergency-driven responses given the fact that the increase was only observed in response to the pandemic rather than during normal times.

Since ABHR was found to be effective in improving hand hygiene in health care [17], WHO has been advocating to improve “system change,” the first element of multi-modal improvement strategy, including local ABHR production at health-facility level. ABHR enables hand hygiene to be performed at point of care in healthcare setting, and has been shown to be effective in resource limited settings where access to soap and water is limited [18, 19], Therefore, access to ABHR is a key to successful hand hygiene promotion in LMICs. Historically, the needs for ABHR also surged during outbreaks, such as Ebola virus diseases in West Africa [20]. This scoping review has revealed that ABHR is now manufactured at factory level, using locally available ingredients, in addition to previously reported local ABHR production mainly led by pharmacists at health-facility level. Both types of local ABHR production were likely promoted during the COVID-19 pandemic, as studies relevant to the COVID-19 pandemic accounted for more than half of the articles included in this review.

However, more than half of the included articles were single-year studies, mainly cross-sectional studies, and most funded studies were supported by grants from HICs, risking the sustainability of local ABHR production, particularly given the post-COVID-19 pandemic period. The scoping review also reaffirms that pharmacists remain as a focal point for health-facility level production, and though some reported district-level approach, engaging multiple sites [21, 22]. the scalability and task-shifting may be a challenge. The predominance of pharmacist-led production confirms that existing pharmaceutical infrastructure remains central to implementation, yet scaling beyond institutional pilots is limited.

Quality control emerged as a persistent and critical gap. The 2011 WHO global assessment identified quality control as a major barrier [9], and this review reconfirms that challenge. Although a diversity of formulations and formats is now being produced, fewer than half of the studies conducted efficacy or organoleptic evaluations. Among those that did, reporting was often incomplete and rarely benchmarked against international standards. Concerns regarding the quality of ABHR available on the market, particularly during the COVID-19 pandemic, further highlight the need for robust, standardized quality assurance mechanisms [23–25]. Strengthening regulatory capacity, establishing national or regional quality-control laboratories, and implementing routine monitoring frameworks are essential to safeguard product quality, protect patient safety, and support national IPC programme goals. Improved reporting of local production efforts would also facilitate benchmarking, guide targeted investment, and ensure alignment with global action plans for hand hygiene [26, 27].

This review has limitations. First, only English-language, peer-reviewed studies were included; relevant evidence in the grey literature or in other languages may have been missed. Nevertheless, the systematic search across major databases supports the robustness of the evidence identified. Second, authors of primary studies were not contacted, and some details, particularly concerning production processes, cost, and quality-control approaches may be incomplete. Further research, ideally using global or regional surveys of IPC practitioners and ABHR producers, is warranted to capture operational realities, including financial structures such as external grant funding and domestic health finance, sustainability and scalability considerations, and organizational models at facility, district, and national levels.

## Conclusions

Local ABHR production has expanded, particularly during the COVID-19 pandemic, yet remains inconsistently documented and largely dependent on short-term, externally funded initiatives. This scoping review highlights persistent challenges related to sustainability, scalability, and quality assurance, including limited reporting of efficacy testing and reliance on facility-based pharmacist-led production. Strengthening long-term, in-country capacity for quality-controlled ABHR production will require integration into national IPC programmes, incorporation into essential-medicine and supply-chain systems, establishment of robust monitoring and reporting mechanisms, and sustained regulatory oversight to ensure product safety and reliability.

## Supplementary Information

Additional file1 (DOCX 19 KB)

## Data Availability

All data and materials generated during the current study are available from the corresponding author on reasonable request.

## Abbreviations

ABHR: Alcohol-based handrub
HICs: High-income-countries
IPC: Infection prevention and control
JBI: Joanna Briggs Institute
LMICs: Low- and middle-income countries
PRISMA-ScR: Preferred reporting items for systematic reviews and meta-analyses extension for scoping reviews
SDGs: Sustainable development goals
UHC: Universal health coverage
UNICEF: United Nations Children’s Fund
WaSH: Water, sanitation and hygiene
WHO: World Health Organization

## References

[1] World Health Organization. The selection and use of essential medicines 2023: web annex A: World Health Organization Model List of Essential Medicines: 23rd list (2023) [Internet]. World Health Organization; 2023 [cited 2025 Nov 7]. Available from: https://iris.who.int/items/0bd7f7b4-0c31-47a4-adb2-69cb4f38c0da

[2] World Health Organization. WHO Guidelines on Hand Hygiene in Health Care: First Global Patient Safety Challenge: Clean Care is Safer Care. Geneva, Switzerland: World Health Organization; 2009. p. 262. 23805438

[3] World Health Organization. Guide to local production: WHO-recommended handrub formulations. 2010 [cited 2025 Apr 25]; Available from: https://iris.who.int/handle/10665/332005

[4] World Health Organization, United Nations Children’s Fund. WASH in health care facilities: global baseline report 2019 [Internet]. World Health Organization; 2019 [cited 2025 Sept 5]. Available from: https://iris.who.int/handle/10665/311620

[5] United Nations. The 17 GOALS, Sustainable Development [Internet]. [cited 2025 Nov 29]. Available from: https://sdgs.un.org/goals

[6] Tartari E, Bellissimo-Rodrigues F, Pires D, Fankhauser C, Lotfinejad N, Saito H, et al. Updates and future directions regarding hand hygiene in the healthcare setting: insights from the 3rd ICPIC alcohol-based handrub (ABHR) task force. Antimicrob Resist Infect Control. 2024;13(1):26. 38424571 10.1186/s13756-024-01374-9 PMC10905912

[7] Brauer M, Zhao JT, Bennitt FB, Stanaway JD. Global access to handwashing: implications for COVID-19 control in low-income countries. Environ Health Perspect. 2020;128(5):057005. 32438824 10.1289/EHP7200 PMC7263456

[8] Benkeser D, Díaz I, Luedtke A, Segal J, Scharfstein D, Rosenblum M. Improving precision and power in randomized trials for COVID‐19 treatments using covariate adjustment, for binary, ordinal, and time‐to‐event outcomes. Biometrics. 2021;77(4):1467–81. 32978962 10.1111/biom.13377 PMC7537316

[9] Bauer-Savage J, Pittet D, Kim E, Allegranzi B. Local production of WHO-recommended alcohol-based handrubs: feasibility, advantages, barriers and costs. Bull World Health Organ. 2013;91(12):963–9. 24347736 10.2471/BLT.12.117085 PMC3845264

[10] World Health Organization. WHO research agenda for hand hygiene in health care 2023–2030: summary [Internet]. Geneva: World Health Organization; 2023. Available from: https://apps.who.int/iris/handle/10665/367527

[11] Allegranzi B, Tartari E, Kilpatrick C, Storr J, Bellare N, Bana J, et al. WHO global research agenda for hand hygiene improvement in health care: a Delphi consensus study. Infect Control Hosp Epidemiol. 2025;46(5):449–64. 40109269 10.1017/ice.2025.32 PMC7617569

[12] Tricco AC, Lillie E, Zarin W, O’Brien KK, Colquhoun H, Levac D, et al. PRISMA extension for scoping reviews (PRISMA-ScR): checklist and explanation. Ann Intern Med. 2018;169(7):467–73. 30178033 10.7326/M18-0850

[13] Peters MDJ, Marnie C, Tricco AC, Pollock D, Munn Z, Alexander L, et al. Updated methodological guidance for the conduct of scoping reviews. JBI Evid Synth. 2020;18(10):2119–26. 33038124 10.11124/JBIES-20-00167

[14] The World Bank. World Bank Country and Lending Groups [Internet]. [cited 2025 Nov 29]. Available from: https://datahelpdesk.worldbank.org/knowledgebase/articles/906519-world-bank-country-and-lending-groups

[15] Saito H, Tartari E, Garlasco J. A scoping review of local production of alcohol based handrub. 2025 Feb 7 [cited 2025 Nov 8]; Available from: https://osf.io/3ka54

[16] European standard EN 1500. Chemical disinfectants and antiseptics. Hygienic handrub. Test method and requirements. European Committee for Standardization, Brussels; 2013.

[17] Pittet D, Hugonnet S, Harbarth S, Mourouga P, Sauvan V, Touveneau S, et al. Effectiveness of a hospital-wide programme to improve compliance with hand hygiene. The Lancet. 2000;356(9238):1307–12. 10.1016/s0140-6736(00)02814-2 11073019

[18] Allegranzi B, Sax H, Bengaly L, Riebet H, Minta DK, Chraiti MN, et al. Successful implementation of the World Health Organization hand hygiene improvement strategy in a referral hospital in Mali, Africa. Infect Control Hosp Epidemiol. 2010;31(2):133–41. 20017633 10.1086/649796

[19] Saito H, Inoue K, Ditai J, Wanume B, Abeso J, Balyejussa J, et al. Alcohol-based hand rub and incidence of healthcare associated infections in a rural regional referral and teaching hospital in Uganda (‘WardGel’ study). Antimicrob Resist Infect Control. 2017;28(6):129. 10.1186/s13756-017-0287-8 PMC5745753 29299303

[20] Vetter P, Dayer JA, Schibler M, Allegranzi B, Brown D, Calmy A, et al. The 2014–2015 Ebola outbreak in West Africa: Hands On. Antimicrob Resist Infect Control. 2016;5(1):17.

[21] Ishida K, Lozier M, Medley AM, Trinies V, Hug C, Ripkey C, et al. Changes in access to alcohol-based hand rub and hand hygiene adherence among healthcare workers after a hand rub production and distribution program in rural uganda before and during the COVID-19 pandemic. Am J Trop Med Hyg. 2024;111(6):1343–52. 39293424 10.4269/ajtmh.24-0040 PMC11619488

[22] Tusabe F, Ishida K, Ocitti F, Yapswale S, Kesande M, Isabirye H, et al. Cleaning and Disinfection Practices of Reused Alcohol-Based Hand Rub Containers in Health Care Settings: Evidence from Five Rural Districts in Uganda. Am J Trop Med Hyg. 2025;18:tmpd240189. 10.4269/ajtmh.24-0189 PMC12062669 39965207

[23] Selam MN, Habte BM, Marew T, Bitew M, Getachew T, Getachew S, et al. Evaluation of quality and antimicrobial efficacy of locally manufactured alcohol-based hand sanitizers marketed in Addis Ababa, Ethiopia in the era of COVID-19. Antimicrob Resist Infect Control. 2022;11(1):126. 36209192 10.1186/s13756-022-01163-2 PMC9547578

[24] Saab Y, Zgheib R, Nakad Z, Khnayzer RS. Determination of volatile impurities and ethanol content in ethanol-based hand sanitizers: compliance and toxicity. Toxicol Rep. 2024;13:101709. 39247052 10.1016/j.toxrep.2024.101709 PMC11379663

[25] Berkkan A, Ulutaş OK. Evaluation of Alcohol Content of Cologne Products in the Turkish Market Amid the COVID-19 Pandemic. Gazi Medical Journal [Internet]. 2020 [cited 2025 Nov 30];31(3A). Available from: https://gazimedj.com/articles/doi/gmj.2020.121

[26] World Health Organisation. Global action plan and monitoring framework on infection prevention and control (IPC), 2024–2030. [Internet]. World Health Organisation; 2023 [cited 2025 Nov 29]. Available from: https://cdn.who.int/media/docs/default-source/integrated-health-services-(ihs)/ipc/ipc-global-action-plan/who_gampf_w_annexes.pdf?sfvrsn=aef723f7_3

[27] MacLeod C, Braun L, Caruso BA, Chase C, Chidziwisano K, Chipungu J, et al. Recommendations for hand hygiene in community settings: a scoping review of current international guidelines. BMJ Open. 2023;13(6):e068887. 37344109 10.1136/bmjopen-2022-068887 PMC10314431

[28] Nigro F, Tavares M, Sato De Souza De Bustamante Monteiro M, Toma HK, Faria De Freitas ZM, De Abreu Garófalo D, et al. Changes in workflow to a university pharmacy to facilitate compounding and distribution of antiseptics for use against COVID-19. Res Soc Adm Pharm. 2021;17(1):1997–2001. 10.1016/j.sapharm.2020.09.016 PMC7527879 33023831

[29] Müller SA, N’Guessan M, Wood R, Landsmann L, Rocha C, Kouame BJ, et al. Effectiveness and sustainability of the WHO multimodal hand hygiene improvement strategy in the University Hospital Bouaké, Republic of Côte d’Ivoire in the context of the COVID-19 pandemic. Antimicrob Resist Infect Control. 2022;11(1):36. 35177123 10.1186/s13756-021-01032-4 PMC8851710

[30] Caniza MA, Dueñas L, Lopez B, Rodriguez A, Maron G, Hayden R, et al. A practical guide to alcohol-based hand hygiene infrastructure in a resource-poor pediatric hospital. Am J Infect Control. 2009;37(10):851–4. 19796845 10.1016/j.ajic.2009.05.009

[31] Pfäfflin F, Tufa TB, Getachew M, Nigussie T, Schönfeld A, Häussinger D, et al. Implementation of the WHO multimodal hand hygiene improvement strategy in a university hospital in Central Ethiopia. Antimicrob Resist Infect Control. 2017;6(1):3. 28070310 10.1186/s13756-016-0165-9 PMC5217264

[32] Gebremicael MN, Skaletz-Rorowski A, Potthoff A, Lemm J, Kasper-Sonnenberg M, Arefaine ZG, et al. Implementing a multimodal intervention using local resources to improve hand hygiene compliance in a comprehensive specialized hospital in Mekelle, Northern Ethiopia. Int J Hyg Environ Health. 2024;259:114389. 38703463 10.1016/j.ijheh.2024.114389

[33] Manaye G, Muleta D, Henok A, Asres A, Mamo Y, Feyissa D, et al. Evaluation of the efficacy of alcohol-based hand sanitizers sold in Southwest Ethiopia. IDR. 2021;14:547–54. 10.2147/IDR.S288852 PMC7887333 33613030

[34] Selam MN, Bayisa R, Ababu A, Abdella M, Diriba E, Wale M, et al. Increased production of alcohol-based hand rub solution in response to COVID-19 and fire hazard potential: preparedness of public hospitals in Addis Ababa. Ethiopia RMHP. 2020;13:2507–13. 10.2147/RMHP.S279957 PMC7667177 33204191

[35] Selam MN, Bayisa R, Ababu A, Abdella M, Diriba E, Wale M, et al. Adequacy of alcohol-based handrub solution production practice in response to COVID-19 in public hospitals found in Addis Ababa, Ethiopia: a multicentered cross-sectional study. J Pharm Policy Pract. 2021;14(1):39. 33934722 10.1186/s40545-021-00321-y PMC8088824

[36] Müller SA, Diallo AOK, Wood R, Bayo M, Eckmanns T, Tounkara O, et al. Implementation of the WHO hand hygiene strategy in Faranah regional hospital, Guinea. Antimicrob Resist Infect Control. 2020;9(1):65. 32410673 10.1186/s13756-020-00723-8 PMC7227248

[37] Müller SA, Diallo AOK, Rocha C, Wood R, Landsmann L, Camara BS, et al. Mixed methods study evaluating the implementation of the WHO hand hygiene strategy focusing on alcohol based handrub and training among health care workers. In Faranah, Guinea. Hodges MH, editor. PLoS ONE. 2021;16(8):e0256760. 10.1371/journal.pone.0256760 PMC8389517 34437634

[38] Sharma M, Joshi R, Shah H, Macaden R, Lundborg CS. A step-wise approach towards introduction of an alcohol based hand rub, and implementation of front line ownership- using a, rural, tertiary care hospital in central India as a model. BMC Health Serv Res. 2015;15(1):182. 25924956 10.1186/s12913-015-0840-1 PMC4424507

[39] Khurana S, Singh P, Sinha TP, Bhoi S, Mathur P. Low-cost production of handrubs and face shields in developing countries fighting the COVID19 pandemic. Am J Infect Control. 2020;48(6):726–7. 32276781 10.1016/j.ajic.2020.03.016 PMC7124313

[40] Tulsawani R, Verma K, Kohli E, Sharma P, Meena YS, et al. Anti-microbial efficacy of a scientifically developed and standardized herbal-alcohol sanitizer. Arch Microbiol. 2024;206(2):77. 38270599 10.1007/s00203-023-03805-4

[41] Putri ND, Satari HI, Karyanti MR, Prayitno A, Wicaksana P, Karuniawati A, et al. Antimicrobial activity of homemade WHO ethanol-based hand rub solution in pediatric department, Dr. Cipto Mangunkusumo National Referral Hospital. PI. 2022;62(4):232–6.

[42] Rafizadeh A, Kolahi AA, Shariati S, Zamani N, Roberts DM, Hassanian-Moghaddam H. The danger of the toxicity and inefficacy of alcohol-based hand rubs in Iran during COVID-19: a cross-sectional study. Antimicrob Resist Infect Control. 2023;12(1):42. 37098641 10.1186/s13756-023-01244-w PMC10127170

[43] Ndegwa L, Hatfield KM, Sinkowitz-Cochran R, D’Iorio E, Gupta N, Kimotho J, et al. Evaluation of a program to improve hand hygiene in Kenyan hospitals through production and promotion of alcohol-based Handrub – 2012–2014. Antimicrob Resist Infect Control. 2019;8(1):2. 30622703 10.1186/s13756-018-0450-x PMC6318974

[44] Ochwoto M, Muita L, Talaam K, Wanjala C, Ogeto F, Wachira F, et al. Anti-bacterial efficacy of alcoholic hand rubs in the Kenyan market, 2015. Antimicrob Resist Infect Control. 2017;6(1):17. 28138386 10.1186/s13756-017-0174-3 PMC5264297

[45] Bausch FA, Heller O, Bengaly L, Matthey-Khouity B, Bonnabry P, Touré Y, et al. Building local capacity in hand-rub solution production during the 2014-2016 Ebola outbreak disaster: The case of Liberia and Guinea. Prehosp Disaster Med. 2018;33(6):660–7. 30394244 10.1017/S1049023X18000985

[46] Kachingwe B, Kumpalume P, Khuluza F, Nyirenda K, Matambo E, Mponda J, et al. A Malawian pharmaceutical response to the COVID-19 Pandemic. Mal Med J. 2022;34(1):60–7. 10.4314/mmj.v34i1.11 PMC10230587 37265827

[47] Lee YF, Lai WH, Lee PY, Ting SCY, Nuja IA, Ngian HU, et al. Acceptability and tolerability of alcohol-based hand rubs among health workers and concessionaires in Malaysia during the COVID pandemic: a hospital-wide cross-sectional study using a modified WHO protocol. Int J Environ Health Res. 2024;34(10):3489–502. 38287203 10.1080/09603123.2024.2309324

[48] Fofanah BD, Kamara IF, Kallon C, Kamara R, Nuwagira I, Musoke R, et al. Evaluating the tolerability and acceptability of a locally produced alcohol-based handrub and hand hygiene behaviour among health workers in Sierra Leone: a longitudinal hospital-based intervention study. BMC Health Serv Res. 2024;24(1):940. 39152407 10.1186/s12913-024-11368-3 PMC11329988

[49] De Bruin W, Van Zijl MC, Aneck-Hahn NH, Korsten L. Quality and safety of South African hand sanitisers during the COVID-19 pandemic. Int J Environ Health Res. 2024;34(2):719–31. 36652575 10.1080/09603123.2023.2166020

[50] Tusabe F, Nanyondo J, Lozier MJ, Kesande M, Tumuhairwe O, Watsisi M, et al. Improving access to WHO formulations of alcohol-based hand rub in healthcare facilities: a district-wide approach. Am J Trop Med Hyg. 2023;109(1):191–200. 37188343 10.4269/ajtmh.22-0554 PMC10324005

[51] Gudza-Mugabe M, Magwenzi MT, Mujuru HA, Bwakura-Dangarembizi M, Robertson V, Aiken AM. Effect of handrubbing using locally-manufactured alcohol-based handrubs in paediatric wards in Harare, Zimbabwe. Antimicrob Resist Infect Control. 2017;6(1):8. 28096976 10.1186/s13756-016-0166-8 PMC5225549

