# Supplementary material for: Global landscape of locally produced alcohol-based handrub in health care settings: a scoping review
Source: Antimicrob Resist Infect Control. 2026 May 9;15:90. doi: 10.1186/s13756-026-01757-0 (PMC13326558; doi:10.1186/s13756-026-01757-0)
Supplement: Supplementary file 1 — Additional file1 (DOCX 19 KB) [file 13756_2026_1757_MOESM1_ESM.docx]

Appendix 1: Search Strategy

Database: Embase (Elsevier)

| **#** | **Searches** |
| --- | --- |
| 1 | hand sanitizer'/exp OR ((hand OR hands OR handrub*) NEAR/3 (sanitiz* OR disinfect* OR antiseptic* OR rub OR asepsis OR decontaminat* OR de-germ* OR degerm* OR alcohol-based OR ABHR OR ABHS OR Gel)):ti,ab,kw,de |
| 2 | (production OR manufact* OR formulat* OR prepared OR preparation OR pharmacy-led OR locally-made OR local-capacity):ti,ab,kw,de |
| 3 | (Local* OR regional* OR domestic OR provincial):ti,ab,kw,de |
| 4 | #2 OR #3 |
| 5 | #1 AND #4 |

Database: Medline (OVID)

| **#** | **Searches** |
| --- | --- |
| 1 | exp Hand Sanitizers/ OR ((hand OR hands OR handrub*) ADJ3 (sanitiz* OR disinfect* OR antiseptic* OR rub OR asepsis OR decontaminat* OR de-germ* OR degerm* OR alcohol-based OR ABHR OR ABHS OR Gel)).ti,ab,kw,kf |
| 2 | (production OR manufact* OR formulat* OR prepared OR preparation OR pharmacy-led OR locally-made OR local-capacity).ti,ab,kw,kf |
| 3 | (Local* OR regional* OR domestic OR provincial).ti,ab,kw,kf |
| 4 | 2 OR 3 |
| 5 | 1 AND 4 |

Database: CINAHL (Ebscohost)

| **#** | **Searches** |
| --- | --- |
| 1 | (MH "Hand Sanitizers+") OR (TX (hand OR hands OR handrub*) N3 (sanitiz* OR disinfect* OR antiseptic* OR rub OR asepsis OR decontaminat* OR de-germ* OR degerm* OR alcohol-based OR ABHR OR ABHS OR Gel)) |
| 2 | TX (production OR manufact* OR formulat* OR prepared OR preparation OR pharmacy-led OR locally-made OR local-capacity) |
| 3 | TX (Local* OR regional* OR domestic OR provincial) |
| 4 | S2 OR S3 |
| 5 | S1 AND S4 |
